# Universal health coverage in ‘One ASEAN’: are migrants included?

**DOI:** 10.3402/gha.v8.25749

**Published:** 2015-01-24

**Authors:** Ramon Lorenzo Luis R. Guinto, Ufara Zuwasti Curran, Rapeepong Suphanchaimat, Nicola S. Pocock

**Affiliations:** 1 Universal Health Care Study Group, University of the Philippines Manila, Manila, Philippines; 2 Nuffield Department of Population Health, University of Oxford, Oxford, United Kingdom; 3 International Health Policy Programme, Ministry of Public Health, Nonthaburi, Thailand; 4 Department of Global Health & Development, London School of Hygiene and Tropical Medicine, London, United Kingdom

**Keywords:** migrant health, migrant workers, ASEAN, Southeast Asia, universal health coverage, health financing

## Abstract

**Background:**

As the Association of South East Asian Nations (ASEAN) gears toward full regional integration by 2015, the cross-border mobility of workers and citizens at large is expected to further intensify in the coming years. While ASEAN member countries have already signed the Declaration on the Protection and Promotion of the Rights of Migrant Workers, the health rights of migrants still need to be addressed, especially with ongoing universal health coverage (UHC) reforms in most ASEAN countries. This paper seeks to examine the inclusion of migrants in the UHC systems of five ASEAN countries which exhibit diverse migration profiles and are currently undergoing varying stages of UHC development.

**Design:**

A scoping review of current migration trends and policies as well as ongoing UHC developments and migrant inclusion in UHC in Indonesia, Malaysia, Philippines, Singapore, and Thailand was conducted.

**Results:**

In general, all five countries, whether receiving or sending, have schemes that cover migrants to varying extents. Thailand even allows undocumented migrants to opt into its Compulsory Migrant Health Insurance scheme, while Malaysia and Singapore are still yet to consider including migrants in their government-run UHC systems. In terms of predominantly sending countries, the Philippines's social health insurance provides outbound migrants with portable insurance yet with limited benefits, while Indonesia still needs to strengthen the implementation of its compulsory migrant insurance which has a health insurance component. Overall, the five ASEAN countries continue to face implementation challenges, and will need to improve on their UHC design in order to ensure genuine inclusion of migrants, including undocumented migrants. However, such reforms will require strong political decisions from agencies outside the health sector that govern migration and labor policies. Furthermore, countries must engage in multilateral and bilateral dialogue as they redefine UHC beyond the basis of citizenship and reimagine UHC systems that transcend national borders.

**Conclusions:**

By enhancing migrant coverage, ASEAN countries can make UHC systems truly ‘universal’. Migrant inclusion in UHC is a human rights imperative, and it is in ASEAN's best interest to protect the health of migrants as it pursues the path toward collective social progress and regional economic prosperity.

Guided by the mantra “One Vision, One Identity, One Community,” the 10 member countries of the Association of Southeast Asian Nations (ASEAN) are now gearing toward full regional economic integration by 2015. As laid out in the ASEAN Economic Community Blueprint, the goal is to transform Southeast Asia into ‘a single market and production base, a highly competitive economic region, a region of equitable economic development, and a region fully integrated into the global economy’ which will allow free flow of goods, services, investment, capital, and skilled labor (1). Given these developments, a further increase in population mobility within the region can be expected in the coming years. For example, the regional bloc has developed ‘Mutual Recognition Arrangements’ that seek to harmonize professional qualification standards, regulations, and procedures across ASEAN member states to facilitate the freer movement and employment of qualified and certified personnel such as doctors, nurses, and dentists (2).

Migration, however, is not a new challenge for the ASEAN region. For the past three decades, Southeast Asia has already become one of the world's most dynamic regions, with a huge volume of migrant workers moving both within the region and between ASEAN and the rest of the world (3). In addition to inter- and intra-regional labor migration, other migration trends have been observed in Southeast Asia, such as undocumented or irregular migration (4) and human trafficking, especially of women and children, for forced labor and the sex industry, which reveal migration's most shameful face (5–7). On the contrary, the region's visa-free policy for ASEAN citizens facilitated the high flux of tourists and other types of temporary migrants from one ASEAN country to another (8). More tourists from other regions are also expected to enter ASEAN's premises once the plan to issue a common visa, similar to the European Union's Schengen visa, is implemented (9).

## Health, well-being, and rights of migrants in ASEAN

Unfortunately, while much attention, including in academic literature, has been devoted to the economic benefits and risks of intra-regional labor migration as well as the social costs of irregular migration and human trafficking within ASEAN, the health and well-being of migrants themselves still remain to be examined (10). For decades, the discourse about health and migration has merely focused on issues pertaining to infectious disease spread and border control measures (11), a narrow view that ignores the individual migrant's well-being and dignity.

Various international declarations and policy instruments have already underscored that health is a fundamental human right that should be enjoyed by all people, including migrants (12–14). In particular, the International Convention on the Protection of the Rights of All Migrant Workers and Members of their Families also emphasized migrants’ right to ‘receive any medical care that is urgently required for the preservation of their life or the avoidance of irreparable harm to their health … on the basis of equality of treatment with nationals of the State concerned’ (15). The 2008 World Health Assembly Resolution 61.17 also urged countries to ‘promote migrant-sensitive health policies’ and to ‘devise mechanisms for improving the health of all populations, including migrants’ (16). Finally, the health of migrants was also featured in the World Migration Report 2013 published by the International Organization for Migration. It is the first-ever report of its kind that focused on migrant well-being, thereby placing the migrant at the center of migration discourse (17).

In Southeast Asia, several efforts are also under way to build momentum around the issue of migrant rights and welfare. In 2007, the ASEAN member countries signed the Declaration on the Protection and Promotion of the Rights of Migrant Workers, which laid down the obligations of sending and receiving states in promoting the fundamental rights and dignity of migrant workers and their families (18). Protecting migrants’ rights was also identified as a strategic objective under the ASEAN Socio-Cultural Community (ASCC) Blueprint (19). Unfortunately, neither of the said regional instruments explicitly mentioned Migrants' right to health or health-related obligations of ASEAN member states toward migrant workers and other people on the move.

Now more than ever, addressing migrant health is necessary, as health problems faced by migrants have become increasingly glaring in recent years. For example, HIV–AIDS has been a major concern among migrants in Southeast Asia (20), particularly among migrant workers entering Thailand (21, 22) and ‘Overseas Filipino Workers’ returning or even deported back to the Philippines (23, 24). In addition, limited access to healthcare among migrants has also been featured in recent regional dialogues organized by various intergovernmental organizations (25). Unfortunately, little information is known about other health vulnerabilities commonly experienced by migrants such as occupational hazards, injuries, and chronic non-communicable diseases.

## Worldwide momentum for universal health coverage – are migrants included?

While migration continues to shape the health of Southeast Asians, the global health community is rallying around the goal of achieving universal health coverage (UHC), which is defined by the World Health Organization (WHO) as providing all people with access to needed health services without incurring financial hardship (26). UHC has already been achieved by most developed countries, in particular for their own citizens, and is now being pursued by being pursued by almost one hundred countries (27), including members of ASEAN (28), with varying levels and speed of progress. Furthermore, UHC is now being advocated by the global health community as an intrinsic component of the health goal for the post-2015 development agenda (29). Today, most countries already have or are establishing pre-payment and risk-pooling systems that combine tax-based financing with premium-based social health insurance and veer away from inefficient and expensive ‘fee-for-service’ payment models, all aimed to reduce out-of-pocket expenditures and prevent impoverishment among households (30).

With this timely campaign toward UHC spreading across the world and particularly within the ASEAN region, and given the background of increasing international migration as described earlier, it is legitimate to ask the question, “Are migrants included in UHC in ‘One ASEAN’?” This paper therefore seeks to explore the nexus between migration and UHC in the ASEAN context, and in particular to examine the nature and level of inclusion of migrants in the UHC schemes of various ASEAN countries.

**Fig. 1:**
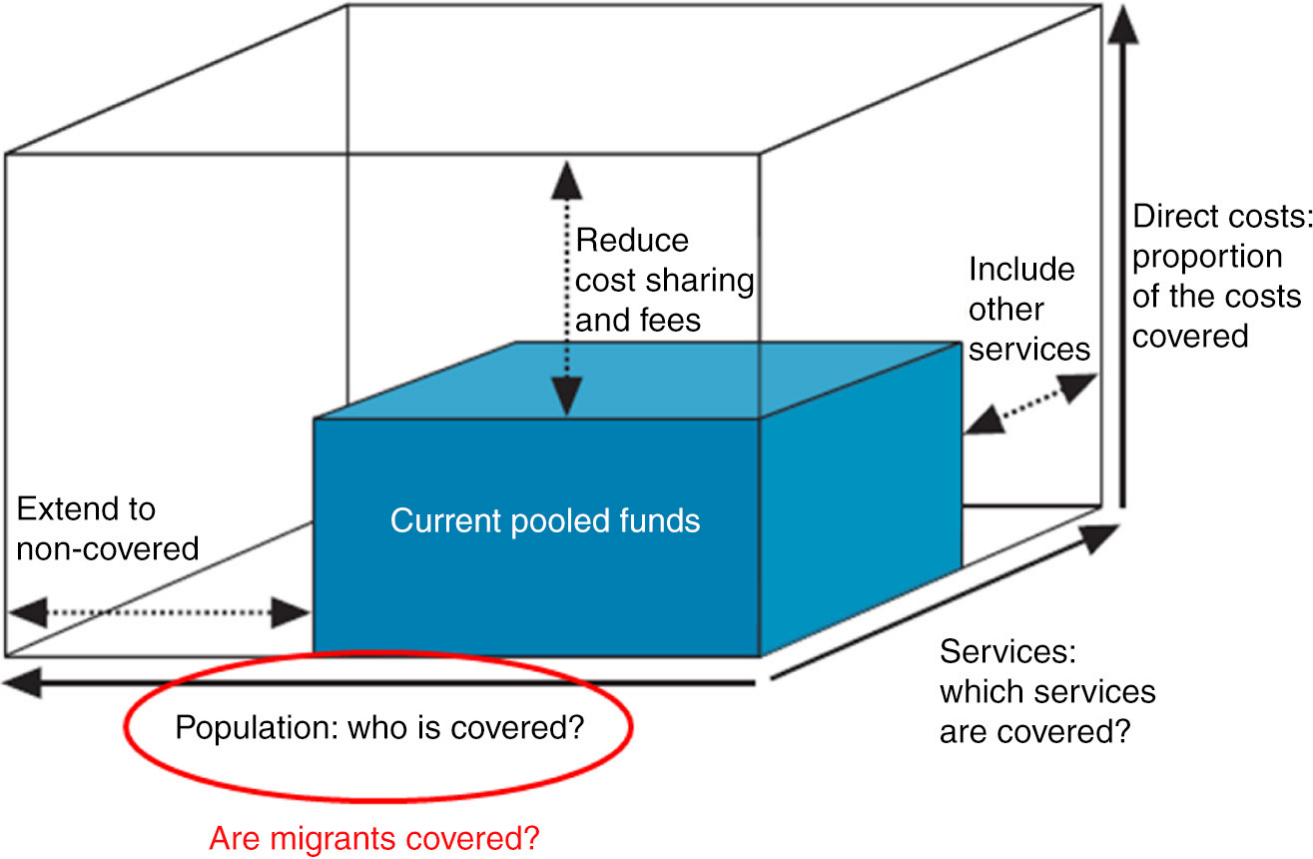
Three dimensions to consider when moving toward universal health coverage, with emphasis on migrant population coverage. Adapted from Ref. (31).

While diverse frameworks have been developed to build a common understanding of UHC, this paper uses the classic UHC cube introduced by the WHO as a guiding framework for analysis (31). The question of whether migrants are considered, enrolled, and covered falls at large under the first dimension of UHC which is population coverage, represented by the x-axis of the cube (Fig. 1).

Nevertheless, this paper attempts to also tackle the other two dimensions – benefit coverage (termed ‘services’ in Fig. 1), which pertains to the range of healthcare services (promotive, preventive, curative, rehabilitative, palliative) that are provided and paid for by the UHC system; and the level of financial protection (termed ‘direct costs’ in Fig. 1), which pertains to the proportion of costs of services covered by the financing scheme. While population coverage is usually given priority first, the two other dimensions are also important for migrants – ideally, in a UHC system, they should at least be enjoying the same basic benefits as well as some level of financial protection (through reduction of cost-sharing resulting in out-of-pocket payments [OPPs]) that are accorded to non-migrants in the countries of origin and destination alike. Furthermore, ideally, migrant-relevant health services such as medical screening and packages for travel- and occupation-related conditions should also be included in the range of benefits covered.

## Methodology

Since migration is a broad term, this paper only focused on international migrants, particularly international labor migrants and undocumented or irregular migrants (In this paper, the two terms are used interchangeably.). Internal migrants, particularly internally-displaced persons as a result of natural calamities or conflicts, are therefore not included in this paper. Nevertheless, UHC reforms should also consider internal migration to ensure people's access to healthcare anywhere within a country's borders (i.e. portability of health benefits).

For the purpose of this analysis, five out of the 10 ASEAN countries were selected – Indonesia, Malaysia, Philippines, Singapore, and Thailand. While a major consideration for the selection is the familiarity with and interest in these identified countries among the authors, these countries also best represent the entire Southeast Asian region in terms of both migration trends and UHC status. Two countries – Indonesia and Philippines – are predominantly sending countries, while Malaysia, Singapore, and Thailand are major destinations for migrant workers. Furthermore, the five countries are at varying stages in the evolution of UHC.

As this topic is a new area of policy and research, a scoping review approach was adopted. Relevant literature, including grey literature such as government policy documents and reports, media articles, as well as publications made by international institutions, published from 2000 to 2014 was reviewed, with the exception of UN resolutions and national laws enacted before 2000. Key terms such as ‘UHC’, ‘health insurance’, ‘Southeast Asia’, ‘migrant’, as well as the names of the five countries were used to search for references in Google, Google Scholar, and PubMed. Grey literature published in the native languages of Indonesia, Malaysia, and Thailand were also searched and provided by authors who are familiar with migration and UHC issues in those countries. Reference sections of retrieved articles were also checked for other relevant sources that were not captured by the aforementioned search engines.

Latest comparable migration- and UHC-related data were compiled and analyzed manually. Current migration trends, such as migrant stocks and flows, and migrant policies and issues in each country were briefly described. This was followed by discussions about ongoing developments in the UHC projects of the five study countries as well as the migrant health-related features of these systems, with emphasis on the three dimensions of the WHO UHC cube when applicable. Gaps, challenges, and opportunities for mainstreaming migrant health into UHC were then identified.

## Results

### Migration trends and policies in ASEAN

Table 1 summarizes the diverse migration profiles among the five ASEAN countries. Among the receiving countries, Thailand has the largest absolute number of in-migrants, followed by Malaysia and Singapore. However, as a percentage of population, migrants make up almost half of Singapore's total population, compared to only 8.3% in Malaysia and 5.6% in Thailand. It should be noted that, while this paper focuses on inclusion of migrant workers and undocumented migrants, these values also include foreign permanent residents (especially in the case of Singapore) as well as refugees and other migrant categories.

**Table 1:** Migration trends in five ASEAN countries

Parameter	Description	Indonesia	Malaysia	Philippines	Singapore	Thailand	Sources of data
General trend	Depends on the percentage out- or in-migration of total population	Sending	Receiving	Sending	Receiving	Receiving	
Out-migration	Stock estimate of citizens overseas (most recent update available) Includes permanent, temporary, and irregular migrants; tourists not included	2,992,550–6,000,000 in 2013	Estimated at 1 million in 2010	10,489,628 as of December 2012 in 218 countries and territories	192,300 in 2011	1,006,051 as of beginning of 2010	Indonesia: (32) Malaysia: World Bank. Malaysia Economic Monitor: Brain Drain. Kuala Lumpur: World Bank Malaysia; April 2011. Available from: http://www-wds.worldbank.org/external/default/WDSContentServer/WDSP/IB/2011/05/02/000356161_20110502023920/Rendered/PDF/614830WP0malay10Box358348B01PUBLIC1.pdf [cited 10 May 2014] Philippines: Commission on Filipinos Overseas. Stock Estimate of Overseas Filipinos (as of December 2012). Manila: CFO; 2012. Available from: http://www.cfo.gov.ph/images/stories/pdf/StockEstimate2012.pdf [cited 10 May 2014] Singapore: National Population and Talent Division of the Prime Minister's Office, Singapore Department of Statistics, Ministry of Home Affairs, and Immigration & Checkpoints Authority. Population in Brief 2011. Singapore: September 2011 Thailand: Huguet JW, Chamratrithirong A. Thailand Migration Report 2011 – Migration for development in Thailand: Overview and tools for policymakers. Bangkok: IOM Thailand; 2011
	As a percentage of total population	1.24–2.49	3.54	11.23	3.79	1.52	Total population data (2010 estimates) from the United Nations, Department of Economic and Social Affairs, Population Division (2012). World population prospects: the 2012 revision. Available from: http://esa.un.org/unpd/wpp/Excel-Data/population.htm [cited 24 June 2014]
Outward labor migration	Number of deployed workers or skilled migrants overseas in a given year (most recent estimate)	512,168 deployed in 2013	Approximately 330,000 skilled migrants in 2010	1,802,031 deployed in 2012	No information	147,623 deployed in 2011	Indonesia: National Agency for the Protection and Placement of Indonesian Migrant Workers (BNP2TKI). Available from: https://docs.google.com/file/d/0B9zVxTquSWwdQnUwVFlreHI0Y0NaT29JSDBFVnpOS3l1ZkJZ/edit Malaysia: World Bank. Malaysia Economic Monitor: Brain Drain. Kuala Lumpur: World Bank Malaysia; April 2011. Available from: http://www-wds.worldbank.org/external/default/WDSContentServer/WDSP/IB/2011/05/02/000356161_20110502023920/Rendered/PDF/614830WP0malay10Box358348B01PUBLIC1.pdf [cited 10 May 2014] Philippines: (44)
							Thailand: Ministry of Labor, Department of Employment, (2011) as cited in (45)
In-migration	International migrant stock at mid-year, 2013 Includes migrant workers, undocumented migrants, refugees, asylum-seekers, students, and other groups of foreign nationals residing in the country (B) Foreign-born (C) Foreign citizens (R) includes refugees	295,433 (C,R)	2,469,173 (B,R)	213,150 in 2013 (C,R)	2,323,252 (B)	3,721,735 (B,R)	(42)

When it comes to the sending countries, the Philippines reported the largest migrant stock overseas, with nearly 11% of its total population living or working outside of the country. The total includes permanent, temporary, as well as irregular migrants. However, Indonesia is facing difficulties in tracking their migrant flow and arriving at more precise estimates (32) hence the wide estimated range of 3–6 million Indonesians abroad, which nevertheless still reflects a considerably huge out-migration.

Among the three main receiving countries, demand for migrant labor remains high. In Singapore and to an extent, in Malaysia, migrants span the entire skill spectrum, with high-skilled migrants in knowledge industries at one end, and low-skilled migrants concentrated in sectors such as construction, manufacturing, the marine industry, domestic work (house help) and the service sector at the other. Because of the language requirement, there are arguably fewer high-skilled migrants in Thailand; however, its economy remains highly dependent on low-skilled labor, which contribute an estimated 7–10% of the total value of industry, and 4–5% that of agriculture (33).

Despite continuing high demand for migrant labor, all three receiving countries use restrictive policies to varying extents to discourage the use of migrant labor and to prevent permanent settlement of migrant workers. In Singapore for example, employers must pay worker levies to the government (higher for low-skilled than high-skilled workers) as well as SGD 5,000 (USD 4,010) in security bonds (money is returned upon repatriation of worker), and must adhere to sector-specific dependency ceilings. For instance, construction companies are required to hire at least one local worker for every seven foreign workers (34). ‘Foreign workers’ (as opposed to ‘foreign talents’ which refer to highly-skilled workers) are usually hired on short-term contracts (1–2 years).

In countries with large numbers of irregular migrants such as Thailand and Malaysia, intermittent crackdowns, raids on migrant workplaces, and deportations are common, particularly during times of economic or political crises (35). ASEAN countries also deploy migrant labor policy as a foreign policy tool. For example, Indonesia and Cambodia impose periodic bans on sending domestic workers (i.e. house helpers) to Malaysia in response to poor treatment by employers.

From a sending country's perspective, the Philippines had a long history as a sending country especially since the 1970s when labor migration became a centerpiece program in order to address massive unemployment. In 2013 alone, remittances sent by OFWs to families left behind in the Philippines totaled at nearly USD 22.9 billion or 8.4% of the country's gross domestic product (GDP) (36).

Cognizant of the importance of OFWs in Philippine society, the country has through the years developed a sophisticated suite of policies and programs designed to advance their rights and welfare (37), including minimum employment standards for compliance by foreign employers; a social security system that OFWs may register into – the Overseas Workers Welfare Administration (OWWA), considered the first and biggest migrant welfare fund in the world (38); and an extensive network of labor attaches deployed to Philippine embassies and consulates in receiving countries mandated to provide necessary assistance, to name a few. Furthermore, the country has been lauded for its extensive use of bilateral labor agreements that lay down obligations of both the Philippines and the receiving country in order to facilitate the orderly deployment of OFWs as well as to ensure migrant protection (39).

Today, Indonesia, the other major sending country in ASEAN, is also beginning to institute similar policies to protect its citizens overseas. For example, the National Agency for the Placement and Protection of Indonesian Migrant Workers (BNP2TKI) was established in 2006 to oversee the deployment of labor migrants and provide direct welfare and protective services. However, irregular migration remains a huge challenge especially for Indonesia. For example, BNP2TKI reported that in the period of 2006–2012, there were about 4 million Indonesian migrant workers overseas, while the number of undocumented Indonesian migrant workers was estimated to be two to four times higher (40). It was even suggested that the Indonesia–Malaysia migration corridor could be the second largest in the world, surpassed only by that between Mexico and the United States (41).

In terms of intra-regional migration within ASEAN, Malaysia remains the top destination for Indonesians (35% of its overseas citizens) (42) and Filipinos (686,547 deployed as of 2012) (43), while Singapore ranks third in terms of countries with newly hired and re-hired OFWs from the Philippines in 2012 (44). Nonetheless, the Philippines still sends more OFWs to the Middle East than to its neighboring Southeast Asian countries. Meanwhile, as of 2012, roughly 2.5 million of Thailand's low skilled migrants hailed from neighboring countries Cambodia, Laos, and Myanmar, with 1.5 million comprising of either family members of registered migrant workers or undocumented workers (45).

### Migrant inclusion in UHC among ASEAN countries

#### Receiving countries

Among receiving countries, Thailand's government spends comparatively more as a percentage of total health expenditure (75.5%) than Malaysia (55.2%) and Singapore (31%), and has lower OPPs than either country (13.5%) (Table 2). Nevertheless, all three countries have either claimed or been reported to have achieved UHC according to their respective definitions, which in general pertains to healthcare coverage at least for their own citizens (46–48). Thailand and Malaysia adopts a predominantly tax-based financing model, with those employed covered through payroll taxes while the rest of the population, including the poor and the informal sector, through general taxation. Malaysia however has long been considering to shift from a tax-financed system to a social health insurance (49), which could either be an opportunity or further threat to migrant inclusion in UHC if this aspect is not addressed early in the discourse. On the contrary, Singapore's UHC system is financed through medical savings, taxes, and premiums collected through its voluntary scheme for catastrophic illnesses, as described later.

**Table 2:** Health financing among the five ASEAN countries

	OPP as% total expenditure on health, 2012^a^	Total expenditure on health as% of GDP, 2011^b^	General government expenditure on health as% of total expenditure on health, 2011^b^
Indonesia	45.3	2.7	34.1
Malaysia	35.6	3.8	55.2
Philippines	52	4.1	33.3
Singapore	58.6	4.6	31
Thailand	13.1	4.1	75.5

a World Bank (2014). The World Bank DataBank. Available from: http://databank.worldbank.org/data/home.aspx [cited 5 July 2014].

b World Health Organization (2014). Global Health Observatory. Available from: http://www.who.int/gho/en/ [cited 3 August 2014].

#### Thailand

Thailand's National Health Act of 2002 (50) mandated that all Thai citizens not covered by existing schemes for civil servants (Civil Servant Medical Benefit Scheme or CSMBS) and formal private sector employees (Social Security Scheme or SSS) are entitled to the Universal Coverage Scheme (UCS); today, UCS covers approximately 75% of the total population. Legal migrants in the formal sector are also covered under the SSS. In addition, since 2001, the Compulsory Migrant Health Insurance (CMHI) has been enrolling migrant workers upon conduct of pre-employment health screening. Unlike the UCS, which is under the National Health Security Office, CMHI is administered directly by the Ministry of Public Health. Under the CMHI, health benefits, including outpatient and inpatient care, are linked to the hospital where the migrant was registered and screened (51). Irregular migrants are also allowed to opt into the CMHI, and an insurance package for migrant children up to age 7 years is available with annual fees of THB 365 (USD 12) (52).

There are however limitations to the CHMI. First, in August 2013, the annual premium, which is paid in advance by the employer and then later deducted from the migrant's wage, was raised from THB 1,300 to 2,200 (from USD 40 to 68). If they wish to continue their membership in the scheme, migrant workers also have to pay an additional THB 600 (USD 19) for the compulsory health screening every year, as the law is not clear on who should defray the examination cost. Moreover, in general, the benefits are still not the same as the ones made available to Thai citizens under UCS; examples of services not provided for CMHI members but guaranteed to UCS members include as therapy for psychotic and substance abuse patients, dental prosthesis, hemodialysis, and kidney transplant (53). CMHI is generally not portable as it is linked to the province and hospital where the migrant originally registered. Migrant coverage still needs to be expanded, since, as of August 2013, the scheme has only registered 66,000 out of the 1 million targeted beneficiaries (54).

Additionally, while the CMHI policy is quite open for undocumented migrants to be registered to the scheme, some hospitals may request various documents that can deter undocumented migrants from enrolling in the scheme. For instance, in Samut Prakan and Chiang Mai, a recent study found that hospitals often require at least one official document to purchase insurance, such as a temporary legitimate residence permit (also known as Tor Ror 38/1), a passport, or, for undocumented migrants without these identity documents, an approved document from the employer such as their house registration (55). Reasons for such documentary requirements include concerns that the card will be rented out to other migrant workers (as had occurred in some cases) and a general unease among some providers about selling the card to undocumented migrants – who themselves may not feel confident to approach the hospital and purchase the card (55).

#### Malaysia

Since 2011, Malaysia has been implementing the *Skim Perlindungan Insurans Kesihatan Pekerja Asing* (SPIKPA; Hospitalization and Surgical Scheme for Foreign Workers), the mandatory private medical coverage scheme for all foreign workers. Enforced by the Ministry of Health, all foreign workers are required to take up this compulsory scheme from one of 28 insurance providers (56), with a premium of MYR 120 (USD 34) and a total coverage of MYR 10,000 (USD 2,778) for use of any health services in the public health system (57). The scheme is mandatory for foreign workers in all sectors (premiums paid by employer or worker), but it is optional for house helpers and plantation workers (whose premiums must still be paid by employers). In Sabah however the rules differ slightly – plantation owners are required to pay for the premium for their workers. By the end of 2011, an estimated 1.2–1.4 million out of 1.8 million registered migrant workers were covered by the SPIKPA scheme (58, 59). Unlike Thailand's CHMI, SPIKPA does not allow irregular migrants to opt into the scheme.

In addition, migrant workers are also covered under the Workmen's Compensation Act (WCA), which provides for lump sum payments for death and disability and stipulates regulations on employer payment of medical costs. The scheme however has been criticized as the maximum liability of employers, which is MYR 300 (USD 84) for surgical ward treatment and MYR 250 (USD 70) for operation charges, as well as the maximum compensation, which amounts to only MYR 23,000 (USD 6,388) in the case of permanent disablement, are hugely insufficient (60).

Migrant workers are however not eligible to enroll in another worker protection scheme, the Social Security Organization (SOCSO), which provides insurance coverage against job-related injuries and disabilities, workplace accidents, occupational diseases and death (61). Another scheme, the Employee Provident Fund (EPF), requires mandatory monthly contribution among Malaysian formal sector workers which provides disbursements for medical care; however, registration remains optional for migrant workers. As a default, private sector employers may also opt for private insurance schemes for their workers.

Among those not covered (partially or fully) by any of these financing schemes, Malaysian citizens pay MYR 1 (USD 0.28) for every consultation with a general practitioner and MYR 5 (USD 1.39) with a specialist. However, non-citizens are charged MYR 15 (USD 4.2) and MYR 60 (USD 17), respectively (62). Surgeries and other specialist services incur higher OPPs, although Malaysian patients in third class wards at public hospitals can only be billed up to a maximum of MYR 500 (USD 139), or half of that for those aged 60 or over (58, 63). However, for foreigners including migrant workers, the minimum deposit is MYR 400 (USD 111.1) for third class wards and MYR 800 (USD 222.2) for surgical cases. While the WCA stipulates that medical charges above the maximum employer liability should come from public funds, in practice some migrant workers are left with excessive bills that they cannot pay.

In order to access public health services, migrant workers need to produce their private insurance card at the hospital registration counter, omitting the need for upfront cash payments (58). In practice however, many employers keep migrant's passports and health cards, making it difficult for them to seek treatment (64). Nonetheless, the public sector technically cannot refuse emergency care to those who cannot pay via prepaid insurance or OPPs, including irregular migrants.

#### Singapore

Finally, Singapore's healthcare financing framework adopts what is called “multiple layers of protection,” which combines heavy government subsidies for acute hospital care with contributory schemes for primary care and catastrophic illnesses (popularly known as 3M) (65, 66). The first ‘M’ refers to Medisave, a compulsory individual medical savings account to which employers and employees contribute, and which can be used to pay for medical expenses. MediShield, the second financing mechanism, is a low-cost and voluntary medical insurance scheme for catastrophic expenditures, and is typically used for larger medical bills. Currently, Singaporean citizens are allowed to opt out of this publicly-administered risk pool should they prefer to avail of private insurance. Recent changes to MediShield will make it a compulsory scheme with lifelong protection, making it more progressive (risk pooling across the entire population) than its predecessor. The third scheme, MediFund, constitutes the final safety net for needy Singaporean patients. It is a medical endowment fund set up by the government to cover those who cannot pay medical bills, covering those with lower incomes but also those who earn more but face large bills relative to their income.

Migrants in Singapore, whether high- or low-skilled workers, are not included under the 3M scheme, hence the private coverage options made available for them. Employers of high-skilled workers (registered under the ‘Employment Pass’ permit) are not required to purchase medical insurance, while for Work Permit holders (low-skilled foreign workers) or S-Pass holders (semi-skilled foreign workers), employers are required to purchase a minimum private medical insurance coverage of SGD 15,000 (USD 11,193) per year for inpatient care and surgery, a limit which is easily breached in face of large medical bills. Worse, foreign workers are ineligible for medical subsidies; in excess of what the medical insurance package can cover, employers are then required to bear the full costs of medical treatment.

Additionally, in cases of disputes on medical expenses arising from work-related illness or injury, the Work Injury Compensation Act (WICA) provides for a process through which claims can be made for medical leave wages, medical expenses and lump sum compensation for permanent incapacity or death. Under WICA, employers are mandated to provide their migrant employees with private insurance that is sufficient to meet payouts in case of work-related illness or injury. The amount for medical expenses compensation has been capped at SGD 30,000 (USD 22,386) (67), which can also be easily breached due to high cost of services (68). In addition, because of lengthy WICA claims processing, foreign workers lose income. While they are entitled to medical leave wages, very few actually receive them. Many see their work permit cancelled and are issued with a special pass which allows them to stay in Singapore while their claim is being processed, but not to take up employment. Many workers are thus forced to turn to nongovernmental organizations for support or to take illegal employment. For more serious cases, it is not uncommon for employers to quickly repatriate workers in order to avoid paying for medical treatment (69).

#### Sending countries

As Table 2 shows, the sending countries have similar levels of government spending on health as a proportion of total health spending, although Indonesia spends less on health as a proportion of GDP (2.7%) as compared to the Philippines (4.1%). While conventionally, destination countries are expected to ensure access to healthcare for migrants that they receive, source countries have also begun providing basic health coverage for their outgoing migrants.

#### Philippines

Since 1995, the Philippines’ National Health Insurance Program or PhilHealth has been striving to achieve its mandate of ensuring financial risk protection for all Filipino citizens (70). With the current administration's UHC program, PhilHealth has reported 79% population coverage, with the poorest 9.6 million families now already being subsidized by the national and local government (71). In 2013, the NHIA was amended (72) to pave the way for massive reforms in benefit design and provider-payment mechanisms (such as shift from fee-for-service to case-based payments), as well as in reducing co-payments (such as through a ‘No Balance Billing’ policy for indigents) (73).

As social health insurance, PhilHealth is financed primarily through premiums (for both employed and self-employed) and tax-sourced government subsidies (for indigents, retirees, and pensioners). Part of the premium-based scheme is a separate program for overseas workers, which is now called the Overseas Filipinos Program (OFP) in order to also cover non-working Filipinos abroad, including irregular migrants, immigrants, dual citizens, and international students. Land-based OFWs are required to pay their premiums individually, while for sea-based OFWs (i.e. seafarers), shipping companies share the cost. As of January 2014, annual premium costs PhP 2,400 (USD 55). In 2013, there are 3.14 million paying members under the OFP, which also covers 2.73 million additional dependents, totaling 5.86 million or 7.6% of the total population covered (71). A unique feature of the PhilHealth governing structure is the presence of an OFW representative in its board of directors.

PhilHealth membership is mandatory for OFWs who got hired through the Philippine Overseas Employment Administration (POEA), the agency responsible for facilitating overseas deployment. PhilHealth enrolment is in addition to other requirements that are stipulated in the Migrant Workers and Overseas Filipinos Act of 1995, which also requires overseas employers to purchase the same private health insurance, along with other worker protection measures, being provided for their locally-hired employees. Nonetheless, those who were not able to enroll in PhilHealth prior to departure may register via the website or its collecting partners in selected countries. PhilHealth membership also covers dependents (spouse, children, elderly parents) accompanying the overseas Filipino in the destination country or being left behind in the Philippines. Conversely, despite the Philippines being a predominantly sending country, PhilHealth also allows foreign nationals residing or working in the Philippines to enroll in PhilHealth as individually-paying or employed members, provided that they present an Alien Certificate of Registration.

Utilization of healthcare overseas is also covered by PhilHealth; however, members pay out-of-pocket first to be later reimbursed (in contrast to utilization in the Philippines, in which PhilHealth directly pays the accredited healthcare provider). This system occasionally results in difficulties in reimbursements among migrants who are hospitalized overseas. Plans to enable online filing of claims and to contract primary care physicians abroad to care for covered OFWs remain in the pipeline (74). Furthermore, benefit coverage for hospitalizations overseas remains inadequate, as PhilHealth is using the case rates applied in hospitals based in the Philippines. Such scheme disregards the huge differences in medical care costs between the Philippines and overseas.

Besides PhilHealth, as earlier mentioned, the Philippines has a migrant welfare fund called OWWA. Although not required but highly encouraged, membership in OWWA costs USD 25. OWWA provides a wide range of services, from accident, burial, and disability benefits to medical, repatriation, and livelihood assistance. OWWA was also handling health insurance for OFWs until the function was transferred to PhilHealth in 2005. A major critique of OWWA is that membership expires at the same time as the end of employment contract, and therefore migrant workers cannot anymore receive benefits upon return to the Philippines (75).

#### Indonesia

Indonesia seems to be following in the Philippines’ footsteps both in terms of UHC and migrant protection. In January 2014, Indonesia announced its goal to achieve UHC by 2019 (76). The national health insurance program, called *Jaminan Kesehatan Nasional* (JKN), seeks to unify three main existing yet fragmented schemes: *Jamkesmas*, the government-financed health insurance program for the poor and near poor; *Askes* for civil servants and pensioners; and *Jamsostek* for formal sector workers. Prior to JKN, these three separate schemes only cover 40% of its 240 million population (77). In addition to providing health coverage, *Askes* and *Jamsostek* are also social security schemes that include employment injury, retirement, and death benefits (78).

Similar to PhilHealth, membership in the revitalized JKN is mandatory to all Indonesian citizens, as the three existing schemes failed to enroll the country's significantly huge informal sector. The program is to be funded mostly through premiums paid directly by self-employed and informal sector members, or deducted from wages for those employed either in public or private sector. On the contrary, Indonesia's poor – estimated at 86.4 million – are to be subsidized by the national government. JKN members are entitled to a range of personal health services, including promotive, preventive, curative and rehabilitative services (78).

As early as now, *Badan Penyelenggara Jaminan Sosial* (BPJS), a dedicated agency mandated to implement JKN, is already drawing critique from different corners for various reasons, such as inadequate and uncertain funding, lack of proper planning for health facilities and health workers, and poor information dissemination among the public, to name a few (79–82).

Since JKN is still evolving, it will take time before migrants are deliberately considered, like in PhilHealth's OFP. At present, health benefits are incorporated in the compulsory Migrant Worker Insurance Program, which includes illness, accident, and death coverage (83). Furthermore, like in the Philippines, bilateral agreements with select destination countries such as Malaysia stipulate overseas employers’ obligation to provide private health insurance for workplace accidents and pre-employment medical examinations (84). Despite the existence of such protective policies, implementation gaps remain, such as huge numbers of claims unprocessed by insurance companies and ill-defined coverage and excluded conditions (83, 84).

Finally, as a receiving country, Indonesia allows migrants who have worked for at least 6 months to enroll in JKN. However, foreigners in Indonesia are reluctant to join in the young scheme, identifying unclear conditions and redundant coverage as they are already provided with private health insurance by their employers (85).

## Discussion

### Redefining UHC for migrants

Table 3 summarizes the five ASEAN countries’ UHC developments as well as their migrant-related features. Overall, the five countries are not starting from scratch in terms of considering migrants in their respective health systems; however, all countries remain marred with implementation issues, from migrants still not covered with insurance in Thailand to difficulties in benefit reimbursements in the Philippines. Nonetheless, these countries can certainly do better in terms of enhancing migrant inclusion in UHC, primarily in terms of population coverage, but also in the two other dimensions of the WHO UHC cube framework – benefit coverage and level of financial protection – which are also touched in the succeeding discussion.

**Table 3:** Migrant-inclusive features of UHC in five ASEAN countries

	Receiving countries			Sending countries	
	
Parameter	Thailand	Malaysia	Singapore	Philippines	Indonesia
UHC overall design	Predominantly financed from general taxation for the poor and informal sector (UCS) and civil servants (CSMBS) combined with payroll taxes for those employed (SSS); membership mandated by law	Two-tiered system; public sector covering all Malaysian citizens funded by general taxes, while private sector funded through private health insurance and out-of-pocket spending	An innovative financing system comprised of government subsidies, mandatory premiums paid jointly by employer and employee, voluntary opt-out insurance for catastrophic illness, and government subsidy for the indigent; membership mandated by law	Social health insurance (PhilHealth) financed through premiums paid voluntarily (informal sector), payroll taxes (employed), or subsidy from national government budget from taxes (indigents); membership mandated by law	Social health insurance (JKN) financed through premiums paid voluntarily (informal sector), from payroll taxes (employed), or through subsidy from national government budget from taxes (indigents); membership mandated by law
Ongoing UHC developments/current status and challenges	Already achieved UHC especially for Thai citizens (in terms of population and benefit coverage as well as low out-of-pocket payments)	Already achieved UHC especially for Malaysian citizens; however, shift to social health insurance currently being considered	Already achieved UHC especially for Singaporean citizens (in terms of population and benefit coverage); still high out-of-pocket payments (58%)	79% population coverage; still high out-of-pocket payments (52%); fee-for-service payments shifted to case rates; outpatient packages still need to be rolled out; deadline for UHC set in 2016	UHC just recently rolled out in 2014; deadline for UHC set in 2019
Migrant-inclusive features of UHC	Separate scheme for legal migrant workers (CHMI) which also allows undocumented migrants to opt in; provides access to a comprehensive range of services, including antiretroviral treatment	Enrollment in private medical insurance schemes mandatory for legal migrants to avail of publicly-provided services; Workmen's Compensation Act provides guarantee for employer assistance for death and disability	Low- and semi-skilled migrants required to be enrolled in private health insurance by employers; Work Injury Compensation Act provides guarantee for employer assistance for disability and death	Separate procedure for membership for Overseas Filipinos but integrated with the national pool; covers overseas hospitalization and family members in country of destination or left behind; separate life insurance specific for migrant workers also exists (Overseas Welfare Workers Fund)	Migrant health insurance not yet part of UHC system but incorporated in compulsory Migrant Worker Insurance Program
Current status and challenges facing migrant inclusion in UHC	Annual premiums need to be paid by migrants themselves; benefits less comprehensive than those for Thai citizens	Migrants still need to be included in the government-run UHC system (beyond access to emergency care); higher co-payments charged against migrants; undocumented migrants totally left out	Migrants still need to be included in the 3M framework; insufficient benefits provided by private insurance; implementation problems due to unscrupulous employers and insurers	Difficult expansion to enroll undocumented migrants; benefits still inadequate due to overseas adoption of domestic case rates; delays and difficulties in processing reimbursements	Undocumented migrants remain uncovered with compulsory insurance; claims unprocessed by insurers; ill-defined packages and excluded conditions

Among the receiving countries, Thailand, a middle-income country that has already realized UHC for its citizens, can be rated as having gone the furthest in terms of ensuring migrant inclusion in UHC. Its parallel migrant scheme and the flexibility allowing undocumented migrants to opt into the system indicates that Thailand's progressive view of UHC goes beyond coverage on the basis of citizenship. This broad conceptualization of UHC is still yet to surface in the ongoing global discourse on UHC. There has been much talk about UHC being one of countries’ national responsibilities for the fulfillment of the right to health (86), but such a citizenship-based notion disregards a huge number of non-nationals living or working in a globalized and highly mobile world.

Singapore and Malaysia are two major destination countries that also claimed or were documented to have already achieved UHC. However, the UHC systems existing in these countries clearly pertain to universal coverage for their respective citizens only. There remains a considerable number of migrant workers and undocumented migrants in these two countries who are not covered by health insurance or inadequately covered with limited benefit packages and high co-payments. This is primarily due to their adoption of the private health insurance model for migrants, which is worsened by absence of strong regulation especially towards employers of migrant workers. If migrant coverage will be a criterion in gauging whether UHC has been achieved or not, then it can be concluded that Singapore and Malaysia have, in actuality, not yet realized UHC in the broadest sense.

In all three receiving countries, migrant workers are extremely dependent on employers for registration with authorities, insurance schemes, and health providers, as well as for their general upkeep and maintenance. Without proper monitoring and enforcement, employers can and do try to reduce costs by under-insuring workers or, for irregular migrants, not insuring them at all. The Ministries of Health of Malaysia and Singapore may therefore consider including migrants in their government-run UHC systems for their citizens, or developing a separate yet still government-run scheme such as what Thailand has done or implementing tight regulation should they prefer retaining their mandatory private health insurance models for migrants. Furthermore, in order to provide adequate health and financial protection, benefits should also be raised to a level that is on par with that provided for native workers and that is realistic given the average cost of healthcare overseas.

However, Thailand's CHMI scheme could be improved by ensuring portability within the country and by allowing premiums to be paid by installment, alleviating the financial burden imposed by lump sum payments. The Thai Ministry of Public Health may also ensure that hospitals relax documentary requirements in order to encourage undocumented migrants to purchase the CHMI.

Conversely, even before their respective UHC projects have commenced, the two sending countries, Indonesia and Philippines, have already begun considering health protection for the migrants that they deploy overseas. As earlier described, health insurance is previously embedded in the Philippines’ mandatory migrant welfare fund (OWWA); now it has already been transferred to PhilHealth. However, health protection still remains a part of the compulsory insurance for outgoing Indonesian migrants, and much work needs to be done to ensure its full implementation and hopefully eventual integration with the newly-established UHC system.

Today, the two countries are embarking on massive health financing reforms toward UHC, and migrant health protection is expected to become a key feature of their UHC systems in the near future. While Indonesia's very young UHC system will still have to focus its resources toward covering its non-moving citizens for now, the Philippines, however, provides a template for predominantly sending middle-income countries on ensuring inclusion of outbound migrants in universal coverage. While still facing operational challenges as well as the need for expanding insurance benefits and the proportion of costs covered, PhilHealth already allows overseas portability of insurance, offers benefit packages for conditions that are relevant to migrants such as viral pandemics (i.e. SARS, Influenza A(H1N1), MERS-CoV), and even extends the benefits to migrant families who are accompanying the migrant abroad or are left behind in the Philippines. Such measures demonstrate the need to reimagine UHC as systems that transcend national borders.

### UHC and migrant health as part of ASEAN's social protection agenda

The issue of UHC among migrants is also very much intertwined with the broader discourse on social protection, whose goal is to secure protection for citizens from lack of work-related income, lack of access to healthcare, insufficient family support, and general poverty and social exclusion (87). Since 2012, the International Labor Organization (ILO) has been advocating for the setting of national ‘social protection floors’ which guarantee access to essential healthcare and basic income security for children, unemployed adults, and older persons (88). Social protection for migrants is even emphasized in the United Nations General Assembly Resolution 40/144 on the human rights of individuals who are not nationals of the country in which they live (89).

The ASEAN regional bloc has also expressed commitment to social protection. In addition to provision of accessible healthcare services, the ASCC Blueprint also identified social welfare and protection as a priority, and envisioned putting in place social safety nets to protect citizens from the negative impacts of integration and globalization (19). While the measures laid down in the blueprint, such as mapping of social protection regimes in ASEAN and the establishment of a social insurance system to cover the informal sector, remain a work-in-progress, there is room for building coherence among the related agendas of social protection, migrant welfare, and UHC.

### UHC – including undocumented migrants?

While challenges in providing health coverage for legal migrant workers by both source and destination countries are now being gradually tackled, coverage among undocumented or irregular migrants, including seasonal migrants, one-day or circular migrants (those who move in for a week or months and then back and come again), and stop-over migrants (those who stay for a while before moving to another country), has oftentimes been avoided due to its sensitive political nature. For instance, the ASEAN Declaration emphasized that ‘the receiving states and sending states shall, for humanitarian reasons, closely cooperate to resolve the cases of migrant workers who, through no fault of their own, have subsequently become undocumented’. However, the Declaration also underscored that it does not imply regularization of the situation of migrant workers who are undocumented (18). This poses a challenge as undocumented persons and refugees, who are not included in existing UHC systems, comprise some of the most vulnerable and marginalized migrant subgroups facing higher health risks and therefore requiring greater attention.

ASEAN member countries may also emulate examples of similar regional blocs that have extensive experience in improving access to healthcare among migrants, including undocumented migrants who are not covered with private health insurance, for instance by their employers. For example, while most countries in Europe provide no more than emergency services for undocumented migrants, some countries either provide more services or allow undocumented migrants to opt into national insurance schemes upon meeting certain requirements such as payment of premiums (90). Among the five ASEAN countries, Thailand and Philippines present some progress though, as both countries already allow irregular migrants (inbound and outbound, respectively) to enroll into the migrant arm of their respective UHC systems.

Including irregular migrants in UHC, whether through tax-based, premium-based or other potential forms of financing, will require a deliberate decision to separate the issue of irregular migrant status from people's entitlement to accessing essential healthcare. While this may not be problematic from a public health perspective, such a stance may strike some sensitive chords in other sectors such as those governing migration and labor policies. Covering undocumented migrants may be misconstrued as condoning irregular migration, even if international human rights instruments that guarantee the right to health to all people regardless of migrant status already exist. Given this situation, crucial policy decisions made by agencies from outside the health sector, such as those that deal with overseas labor, immigration issues and diplomatic relations, are critical. Ministries of Health of ASEAN countries therefore must actively negotiate with their counterparts in government to advocate for realizing the health rights of irregular migrants through UHC. Ultimately, the issue of healthcare access regardless of migrant status may require broadening the focus of migration discourse in ASEAN from mere “ASEAN migrant workers” to “ASEAN citizens.”

### Harnessing ASEAN's open dialogue approach to advance migrant health

For almost half a century, ASEAN has nurtured among its member countries a culture of continuous and open dialogue. In fact, the regional bloc is originally conceived as a loose network of countries to function in that manner, until the idea of a more integrated ASEAN community was conceived in 2003. Nevertheless, regional integration, especially economic integration, demands a deeper level of dialogue about shared pressing issues such as migration and health. These issues, however, cannot be resolved overnight – for example, seven years have already passed since the signing of the ASEAN Declaration on the Protection and Promotion of Rights of Migrant Workers and the instrument that will serve as its implementing guideline is still yet to be finalized and approved.

Nonetheless, promoting migrant health has already been recently identified as a priority in the ASEAN Strategic Framework on Health Development (91). Three of the study countries – Indonesia, Philippines, and Thailand – serve as the lead countries in this area of cooperation. In 2012, Indonesia hosted a workshop on migrant health to develop a set of recommendations for increasing access to health services for migrants. Another possible platform where migrant integration in UHC systems can be discussed is the recently-created ASEAN Plus Three (China, Japan, South Korea) UHC Network (92).

Outside of formal ASEAN platforms, member countries may also take the bilateral route and discuss with counterpart countries on how to improve financial coverage and access to healthcare among migrants. In terms of the ASEAN integration, UHC and migrant health nexus, the roles of origin and destination countries are equally important, particularly in the face of health system inequities between neighboring countries. For instance, Thailand has a more developed health system compared to those in Cambodia, Myanmar and Laos, making it an attractive place to access healthcare among migrants. For political and internal security reasons (e.g. disease control), Thailand and the surrounding countries already have established bilateral collaborations between their respective public health systems (93).

Recently, Thailand and Cambodia have signed an MOU to develop border health services to be implemented by designated national task forces. At a July 2013 meeting, the countries agreed to improve the referral system and care for critically ill patients, as well as non-critical case referrals across the border. One proposal involved building ‘sister hospital’ networks of Thai and Cambodian hospitals on both sides of the border to facilitate cross-border referrals (93). There are several other examples of bilateral cooperation of technical expertise sharing, human resource development and infectious disease control between Thailand, Cambodia, Laos and Myanmar, indicating that an inclusive migrant health approach involves close cooperation with neighboring countries. For example, a series of dialogues has already been undertaken to explore how these countries can jointly address health policy, financing, and care delivery issues for migrants crossing the Thai border (94). Challenging as they may seem due to the huge diversity of healthcare financing arrangements among countries, co-financing mechanisms between sending and receiving countries may also be explored.

### Migrant health and UHC – a new research agenda

One of the major challenges faced during the conduct of this review is the dearth of literature examining migrant health in general, and migrant health in connection to UHC or health systems in particular. To date, limited academic and policy research on how migrants access health services in ASEAN countries means that we do not have a full understanding of the health challenges they face throughout the entire migration cycle. Clearly, there is a need to develop a research agenda that examines this nexus of migration and health (95) and to ensure that health systems and UHC are part of it. Furthermore, research at the country level is therefore highly encouraged, and these studies can feed into the broader regional discourse on migrant inclusion in UHC.

Comparisons between countries also pose a challenge due to the diversity of UHC designs, migration profiles, and migrant protection schemes, not to mention the reliability of data on migration. A monitoring and evaluation framework can later be developed to allow a more comprehensive and robust cross-country comparison. Finally, the link between migration and UHC requires transdisciplinary research, as the question of how UHC can be made migrant-inclusive will need inputs not just from the public health and health systems perspective, but also from labor studies, political science, and international affairs, to name a few.

## Conclusions

In the coming years, with the ongoing move toward regional integration, ASEAN will continue to be a highly dynamic and mobile region. Hence, ASEAN countries should capitalize on the momentum built by both ASEAN integration and the UHC agenda in order to build migrant-inclusive health systems. Origin and destination country efforts to improve migrant health coverage are equally important, and there are more ways than one to ensure that migrants are included in UHC.

The reasons for including migrants in UHC in ASEAN countries are many. First and foremost, addressing the health needs of migrants in ASEAN is a matter of human rights and social justice, which are fundamental principles already enshrined in the regional bloc's numerous instruments. Moreover, it is in ASEAN's best interest to protect the health of migrants as it pursues the regional path toward collective social progress and economic prosperity. Indeed, healthy migrants contribute to the advancement of human capital in both sending and receiving countries, thereby creating healthy communities and healthy economies. ASEAN can also take leadership in the ongoing global conversation on the shape of the post-2015 development agenda, particularly the health goal which is most likely to incorporate UHC. Finally, the region can demonstrate to the rest of the world that UHC can and should go beyond health protection on the basis of citizenship, and therefore must ensure the inclusion of non-nationals (96), and that UHC can be reimagined as systems that transcend national borders. Leaving out migrants in the UHC agenda is clearly not ‘universal’ at all, and is therefore a huge step backward from achieving its very goal – access to affordable and quality healthcare for all, anywhere, all the time.
